# Moisture content monitoring in withering leaves during black tea processing based on electronic eye and near infrared spectroscopy

**DOI:** 10.1038/s41598-022-25112-6

**Published:** 2022-12-01

**Authors:** Jiayou Chen, Chongshan Yang, Changbo Yuan, Yang Li, Ting An, Chunwang Dong

**Affiliations:** 1 Liming Vocational University, Quanzhou, 362007 China; 2 Tea Research Institute, The Chinese Academy of Agricultural Sciences, Hangzhou, 310008 China; 3 College of Engineering and Technology, Southwest University, Chongqing, 400715 China; 4 Tea Research Institute, Shandong Academy of Agricultural Sciences, Jinan, 250033 China

**Keywords:** Engineering, Near-infrared spectroscopy, Imaging and sensing

## Abstract

Monitoring the moisture content of withering leaves in black tea manufacturing remains a difficult task because the external and internal information of withering leaves cannot be simultaneously obtained. In this study, the spectral data and the color/texture information of withering leaves were obtained using near infrared spectroscopy (NIRS) and electronic eye (E-eye), respectively, and then fused to predict the moisture content. Subsequently, the low- and middle-level fusion strategy combined with support vector regression (SVR) was applied to detect the moisture level of withering leaves. In the middle-level fusion strategy, the principal component analysis (PCA) and random frog (RF) were employed to compress the variables and select effective information, respectively. The middle-level-RF (cutoff line = 0.8) displayed the best performance because this model used fewer variables and still achieved a satisfactory result, with 0.9883 and 5.5596 for the correlation coefficient of the prediction set (R_p_) and relative percent deviation (RPD), respectively. Hence, our study demonstrated that the proposed data fusion strategy could accurately predict the moisture content during the withering process.

## Introduction

Black tea, which is a non-alcoholic beverage, is preferred by people around the world due to its unique flavor^1^. Despite its popularity, the complicated manufacturing craft involved is unfamiliar to most consumers. Withering is a crucial step in black tea processing, which could affect the quality of finished tea, including taste, aroma and color^2^. Changes in the moisture level is the most important part of evaluating the withering process. Too much moisture in the withering leaves can lead to the decrease in some key components in the subsequent processing steps of black tea. On the contrary, excessive withering will result in the leaves to be crushed during the rolling process^3^. In actual production, the evaluation of withering degree mainly depends on the sensory experience of tea masters. However, this method is not only insufficient to accurately quantify the moisture content of withering leaves, but also it is vulnerable to external factors. Generally, moisture in withered leaves is in the range of 0.58–0.62 (58%–62%), which is considered as moderate withering^4^. When the moisture level is higher than 0.62 or lower than 0.58, it is regarded as insufficient withering and excessive withering, respectively. Hence, the quantitative detection of moisture content is particularly important. Although certain methods including gravimetric oven and moisture analyzer can accurately determine the moisture level, these are time consuming and destructive. Therefore, a rapid and nondestructive method should be developed.

Some researchers have used nondestructive detection technology in agriculture^5–8^. This is especially applicable for the prediction of moisture content in withering leaves. In the study conducted by Liang et al.^9^, 15 features of withering leaves were used to establish a moisture detection model. The R_p_ and the root-mean-square error of prediction (RMSEP) were 0.9314 and 0.0411, respectively. Shen et al.^10^ proposed the use of Elman neural network to predict the moisture content using miniaturized near-infrared spectroscopy and a smartphone, and the results were satisfactory. The E-eye technology could predict the moisture content of withering leaves because the color and texture features change regularly with the decrease in moisture level. Spectral technology could evaluate the moisture content of withering leaves based on changes in the effective features, such as some bands corresponding to the OH-stretching overtone spectra. Although both of these technologies could obtain the information related to the moisture of the withering leaves from different aspects, the application of a single technology produced one sided results: they only collected sample information from one aspect and ignored other types of sample information, thus it was difficult to collect the overall information of tea samples. From a biochemical perspective, fresh tea leaves display slow enzymatic and non-enzymatic reactions during the withering process. Their moisture and chlorophyll content decrease gradually, and the opposite is true for the theaflavin content, thus leading to a significant color change. Remarkably, near infrared spectroscopy can predict the moisture level based on the internal information of withering leaves, such as the OH-stretching overtone spectra, while machine vision technology uses appearance features for this task^11,12^. However, the obtained information of these technologies is relatively independent. Hyperspectral imaging technology has been used to evaluate the moisture content of tea, especially during the withering process, because it can express the image and spectral in-formation at the same time. An et al.^13^ and Wei et al.^14^, and Dong et al.^4^ established satisfactory moisture prediction models for single and accumulative withering leaves, respectively, and realized the visualization of moisture distribution. However, these studies established a moisture evaluation model using spectra data, and did not involve any appearance information. Only a few studies using data fusion strategy have detected the moisture of tea leaves in withering processing. In a recent study^2^, the withering degree was evaluated successfully by the fusion of colorimetric sensing array (CSA), E-eye and NIRS information. Liu et al.^15^ accurately predicted the moisture content using machine vision and NIRS during green tea processing. The above studies showed that a data fusion strategy could yield better prediction results than any single technology; therefore, developing new fusion strategies involving different technologies is an imminent task for the evaluation of tea quality.

In this study, near infrared spectroscopy was used to describe the internal characteristics of withering leaves, because of the stretching vibration of O–H group; The acquired color and texture features are used to express the external information of withering leaves. During the withering process of black tea, the tea color changes from bright green to dark green to grayish green to grayish brown, and the shape of the tea changes from water loss to curly, leading to the change of texture features. And it has been applied in previous studies^16^, so this paper uses the color and texture features of the image and the near infrared spectroscopy technology to detect the horizontal content of black tea during the withering process. During the withering process, the appearance and moisture level of tea samples undergo considerable changes. To accurately reflect these changes, the present study integrated the information from NIRS and E-eye to comprehensively evaluate the moisture content during the withering process. The detailed steps were as follows: (1) Collect the spectral and image information at different withering time points. (2) Extract the color and texture features of tea images. (3) Establish moisture detection models using each single technology. (4) Construct a moisture detection model based on the fusion of both technologies. Compared with the above studies, the obvious advantages of our research could be summarized as follows: (1) More comprehensive information for withering leaves were obtained. (2) More useful feature pieces of information were selected. (3) Different levels of data fusion strategy were applied to improve the performance of the moisture prediction model.

## Materials and methods

### Samples

**Figure 1:**
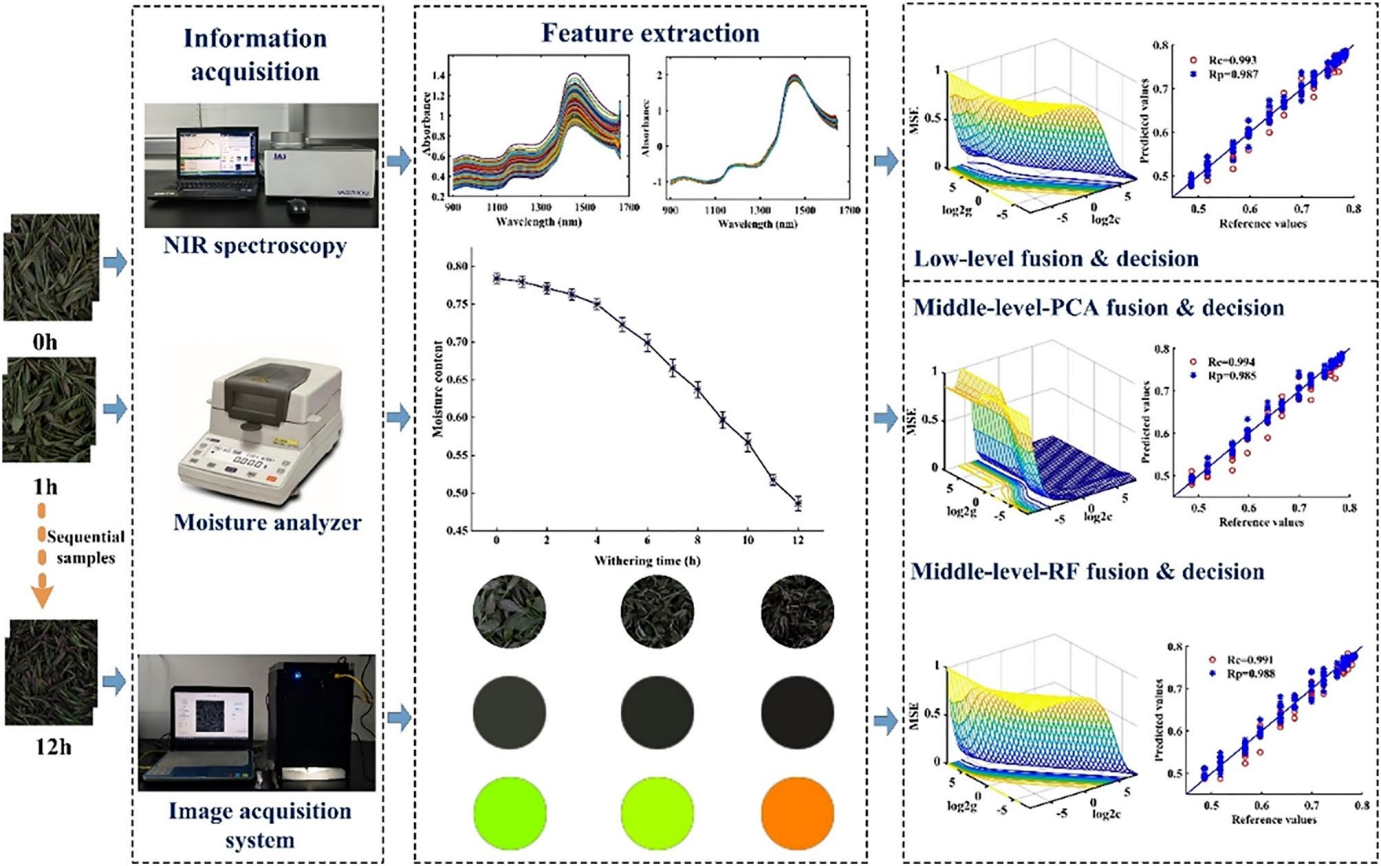
Flow diagram of withering experiment and data analysis.

A total of 25 kg ‘Dahongpao’ fresh tea leaves with ‘one bud, one leaf’ were collected in Shengzhou experimental base of Tea Research Institute of Chinese Academy of Agricultural Sciences for the withering experiment, which was carried out on April 14, 2022. The collection and processing of fresh tea leaves were carried out based on the manufacturing crafts of black tea (GB/T 35810-2018). After recording the initial moisture value, and the spectral and image information of withering leaves, they were placed into a withering through at 3 cm thickness under 30℃ atmosphere and 50% relative humidity. The test lasted for 12 h and several samples were collected every hour. From each sample, 3 g from 5 different locations of all withering samples was taken and placed in a moisture analyzer (MA35M-000230 V1, Sartorious, SHANGHAI YOUYI INSTRUMENT CO.LTD, CHINA) to measure the moisture content of withering leaves, indicating that the obtained samples were uniform and representative. This process was repeated 3 times to obtain 3 moisture values for each withering moment, indicating that the obtained moisture is accurate. Finally, the mean of 3 recorded values was regarded as the moisture at a given withering moment. Remarkably, after the whole withering experiment, the moisture of tea samples decreased from 0.7843 (78.43%) to 0.4863 (48.63%). In addition, other collected samples were placed into NIRS and a self-built image acquisition system, to obtain spectral and image information, respectively. Our experimental data processing strategy is demonstrated in Fig. 1.

### Acquisition of spectral information by NIRS

The IAS-3100 NIR spectrum analyzer (Intelligent Analysis Service Co. LTD) was used to collect the spectra of withering leaves. The applied spectral range was 900–1700 nm, and the spectral accuracy and resolution were ± 1 nm and 4 cm^−1^, respectively. The spectral information was collected at 25℃room temperature in diffuse reflection mode. At each withering moment, 15 parallel samples (approximately 100 g of each sample) were prepared for obtaining the spectral information using the five point sampling method, which could obtain representative withering samples^17,18^. For this method, the center of the diagonal was determined as the central sampling point. Subsequently, four points on the diagonal at equal distances from the central sampling point were selected as other sampling points. These obtained samples were mixed as one withering sample. Finally, all withering leaves were mixed evenly, and the above process was repeated to yield the required withering samples. These samples were placed in a sample pool with a diameter of 130 mm and a height of 50 mm for scanning the spectral information. For each withering sample, the 'scan-stir' process was repeated four times, and the averaged spectra were considered as the sample data. Among them, 195 raw spectra were collected in our withering experiment. These were subjected to the removal of noise bands and standard nor-mal variate (SNV) normalization for preprocessing. Remarkably, SNV could decrease the effects of scattering on tea samples^19^. To improve the performance of the moisture detection model, the optical path correction of raw spectra was necessary.

### Image acquisition using a self-built E-eye system

The self-built E-eye system was mainly composed of a professional industrial camera (FI-S200C-G), a DOME monochrome pure white arc light source (100 lx), and a computer. The professional industrial camera contained a 4 nm low distortion lens. The resolution and exposure time were 1080 × 1080 pixels and 0.09 ms, respectively. The lens of the camera was placed on top of a dark box to determine the constant object distance. During the withering experiment, the same samples were placed into our self-built E-eye system to acquire the image of withering leaves, resulting in a total of 195 sample images. Remarkably, the whole region of the obtained image could be filled with tea samples, which were shown in Fig. 1. The employed image processing software (2014SR149549) was developed using Matlab GUI module to extract the appearance features of withering leaves. In this module, the whole sample image was covered by a rectangle, which had the same size as the sample image. This selected region was used as the region of interest (ROI) to extract color and texture features. Subsequently, 9 color features, including red component mean value (R), green component mean value (G), blue component mean value (B), hue mean value (H), saturation mean value (S), visible light mean value (V), brightness component mean value (L), a component mean value (a), b component mean value (b), and 6 texture features, including average gray value (m), standard deviation (δ), smoothness (r), third moment (μ), consistency (U), and entropy (e), were obtained to establish the moisture detection model. It is worth noting that the HSV space was obtained based on the RGB color space and formulas proposed by a previous study^20^.

### Dimension reduction and data fusion

The collected data information from NIRS and the image acquisition system are multidimensional, and there is some inevitable redundancy in their combined data. In addition, applying multidimensional data not only affect the speed of information collection, but also reduces the running speed of the model. Therefore, to improve the running speed of the model and sample information collection, a data reduction and feature selection strategy was implemented for the collected multidimensional data. Principal component analysis (PCA) is a common variable compression strategy^21^. In contrast to PCA, feature selection is another method to reduce the redundancy of multidimensional data. To accomplish this task and improve the performance of the moisture prediction model, random frog (RF)^22^ and correlation analysis^23^ were performed in this study. Remarkably, the RF strategy could reduce the individual spatial difference by the local sub-population updating strategy and obtain the local optimal solution by the information exchange between each subpopulation^13^. The RF strategy was applied to select effective bands of spectral information, whereas the Pearson correlation analysis was employed to select useful appearance, which was related to the moisture of withering leaves.

Data fusion strategies can obtain effective information from different sensors, thereby improving the effectiveness and comprehensiveness of the collected data. Generally, a data fusion strategy could be divided into three levels, such as low-level (data-level), middle-level (feature-level) and high-level (decision-level)^24^. In contrast to the low- and middle-level fusion strategies, the high-level strategy has not been widely used as it relies too much on the accurate expression of the main information from each sensor^18,25^. Therefore, in this paper, the low- and middle-level fusion strategy were applied to predict the moisture content of withering leaves.

Before establishing the prediction model, the raw data collected using NIRS and E-eye technology were divided into calibration set (143 samples) and prediction set (52 samples) based on the Kennard-Stone method with the ratio of 3:1^26,27^.

### Establishment and evaluation of prediction model

In the SVR algorithm, the raw data are mapped to high-dimensional space based on nonlinear mapping, which has good linear regression characteristics in the feature space. Subsequently, the mapped variables complete linear regression in the feature space and then return to the original space^28^. Importantly, the radial basis function (RBF) was selected as the kernel function, and two parameters including the penalty factor parameter c and kernel function parameter g need to be optimized by cross-verification.

In this research, five evaluation parameters including the correlation coefficient of the calibration set (R_c_), the correlation coefficient of the prediction set (R_p_), the root mean square error of calibration (RMSEC), the root mean square error of prediction (RMSEP) and relative percent deviation (RPD) were determined to evaluate the performance of the moisture prediction model. Importantly, RPD means the ratio of standard deviation to RMSEP. When the values of R_c_, R_p_ were larger and close to 1, the model showed a satisfactory performance. However, the opposite was true for the values of RMSEC and RMSEP. For the RPD values, the higher the value, the better performance of the established model.

### Software

The statistical graphs were drawn using Origin 2021 (OriginLab Corp. Massachu-setts, USA). Other data analyses, including spectra processing, PCA, data fusion and SVR model establishment, were completed using Matlab R2017b software (The Math Work, Inc., Natick, MA, USA).

### Statement of sample collection guidelines

The collection of all fresh leaves was approved by the relevant institution. The collection and processing of samples were carried out based on relevant national standards.

## Results and discussion

### Feature analysis of different sensors

In order to accurately predict the moisture content of withering leaves, their spectral and appearance information were collected to establish the prediction model. These technologies express the information of tea samples from different aspects. Hence, it was necessary to further analyze these sets of data.

#### Response NIR spectra of withering leaves

**Figure 2:**
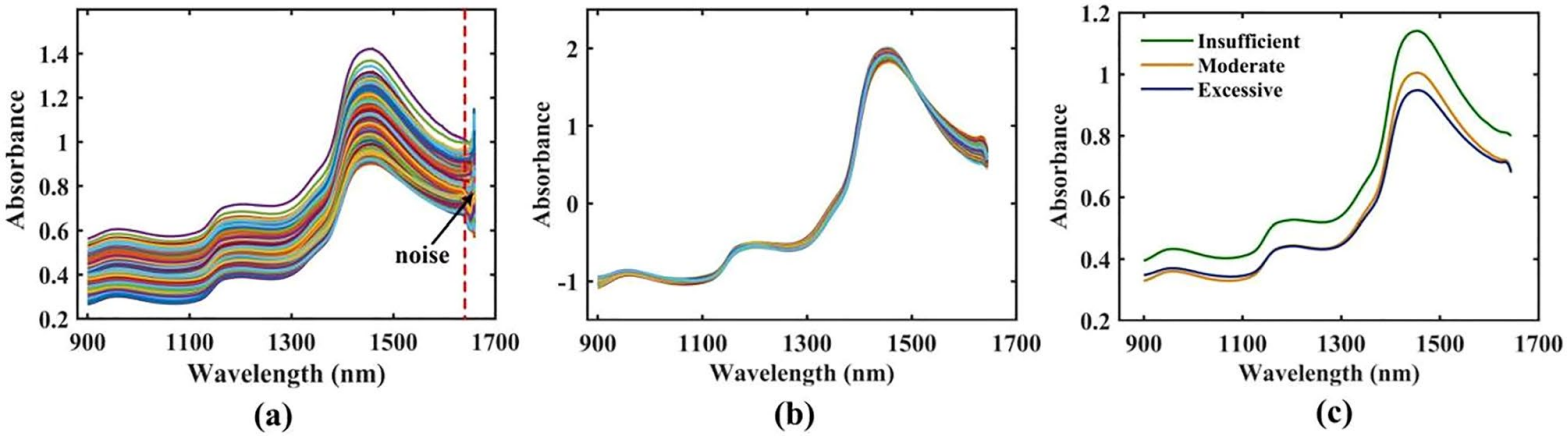
Spectra of all withering leaves acquired from the raw data (**a**); SNV processing data (**b**); and the average spectra of three different withering degrees (**c**).

Figure 2 presents the NIR spectral features of withering leaves. In Fig. 2a, the spectral information of all samples are displayed, and these spectra contain 760 bands. However, the absorbance changes irregularly at the end of raw spectra because of the influence of noise. Therefore, the absorbance of the last 15 bands were eliminated. Subsequently, the raw spectra without noise bands were subjected to SNV preprocessing, and the resulting spectra were displayed in Fig. 2b. SVR models were established using SNV spectral data and the SNV spectral data without noise bands, and the performance of the established SVR models were shown in Table 1. The spectral data without noise bands showed better performance, indicating that these noise bands affected the accuracy of the established moisture detection model. According to the moisture levels in our samples, three withering degrees, such as including insufficient withering, moderate withering and excessive withering, were established, and the average spectral curve of the spectral information for these withering degrees were shown in Fig. 2c. In the figure, the absorbance intensity showed the same trend for different withering degrees. However, there were some differences in the spectral profiles of different withering degrees. For instance, the spectra of insufficient withering leaves showed higher absorbance than the spectra of other withering degrees, a phenomenon also demonstrated by a previous study^2^. In addition, the absorbance intensity near 1450 nm decreased over the withering time. As can be seen in Fig. 2, obvious absorption peaks appear near 965 nm, 1093 nm, 1176 nm and 1450 nm. The absorption peaks near 960 nm and 1093 nm correspond to the second free OH and bound OH-stretching overtone spectra for water^29^. Furthermore, the absorption peak near 1176 nm is attributable to the second CH-stretching overtone spectra for catechins^30^. In addition, the absorption peak around 1450 nm is related to the first OH-stretching overtone spectra for water^31^. The groups corresponding to these absorption peaks provide a theoretical basis for establishing the moisture detection model.

**Table 1:** SVR models for the moisture content of withering leaves using individual data.

Data	No. of variables	Parameter		Calibration sets		Prediction sets		
		c	g	R_c_	RMSEC	R_p_	RMSEP	RPD
NIR (noise)	760	0.25	3.0314	0.9969	0.0081	0.9787	0.0205	4.6861
NIR	745	0.25	3.0314	0.9969	0.0081	0.9788	0.0204	4.7213
E-eye	15	0.3299	0.0272	0.9935	0.0116	0.9823	0.0216	4.6491

#### Color and texture features of withering leaves

As can be seen from Fig. 1, the color of withering leaves changed from bright green to dark green because of the moisture reduction and the transformation of chlorophyll in withering leaves. Experienced tea masters usually evaluate the withering degree by the tactile information of withered leaves, which is related to the texture features of tea samples. To quantify the sensory experience of tea masters, 9 color features, such as R, G, B, H, S, V, L, a, b, and 6 texture features, including m, δ, r, μ, U, and e, were extracted and analyzed. Although these extracted variables could quantify the sensory experience of tea masters, there might be a certain correlation between these features, which could affect the performance of the moisture detection model. Hence, the correlation analysis of the extracted 15 features from the sample image was performed, and the results were demonstrated in Fig. 5. The size and color of the circle indicates the degree of correlation between two variables, where the red color means positive correlation and the blue color indicates negative correlation. In Fig. 5, most variables are clearly correlated with each other. Hence, these color and texture features should be further discussed.

### Feature selection of different sensors

The NIR spectral and image information of tea samples were obtained from multi-dimensional channels, which contained redundancy and would affect the performance of the moisture detection model. Hence, RF algorithm and Pearson correlation analysis were performed to select the effective information for both spectral and image data.

#### Feature selection for NIRS

**Figure 3:**
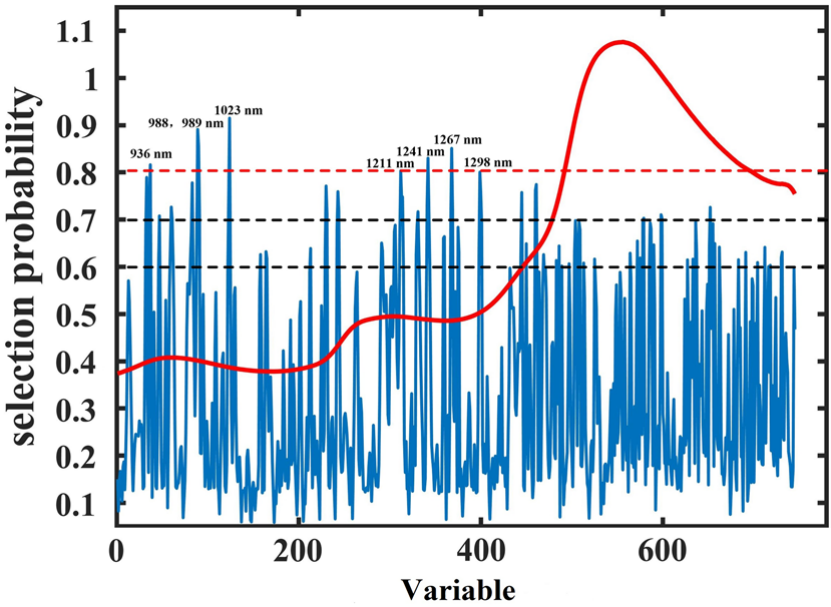
Selection probability of each variable by RF.

**Figure 4:**
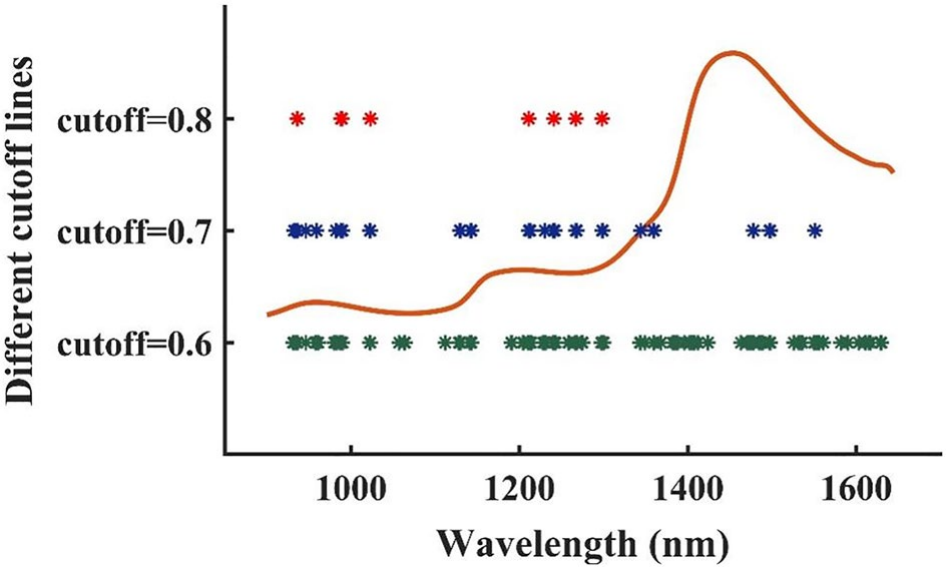
Selected effective features for the spectra data.

For the spectra of withering samples, the RF strategy was applied to retain effective information for the moisture prediction model. In the RF strategy, the number of simulations was determined as 10,000. The latent variables for cross-validation and the number of variables in the initial model for jumping were 15 and 1, respectively. According to the selection probability of each variable, some effective bands were selected. Figure 3 demonstrates the average spectra of all withering samples and the selection probability of each spectral variable. In the figure, the selection probability of different spectral variables displays significant differences. To reduce the redundancy and improve the performance of the moisture prediction model, only the variables with high selection probability were retained. Hence, three cutoff lines of 0.6, 0.7 and 0.8 were determined in this research. If the selection probability of a variable was higher than these predetermined cutoff lines, that variable was selected, otherwise it was eliminated. When the cutoff lines were set as 0.6, 0.7 and 0.8, the selected spectral bands were 101, 33 and 8, respectively. The selected spectral bands were displayed in Fig. 4. Furthermore, the compression rate for cutoff line = 0.6, cutoff line = 0.7 and cutoff line = 0.8 was 86.44%, 95.57% and 98.93%, respectively, indicating that some irrelevant variables were removed from the moisture prediction model. Subsequently, the retained spectral variables based on the three cutoff lines were used for combination with effective appearance features for further analysis.

#### Feature selection for E-eye

**Figure 5:**
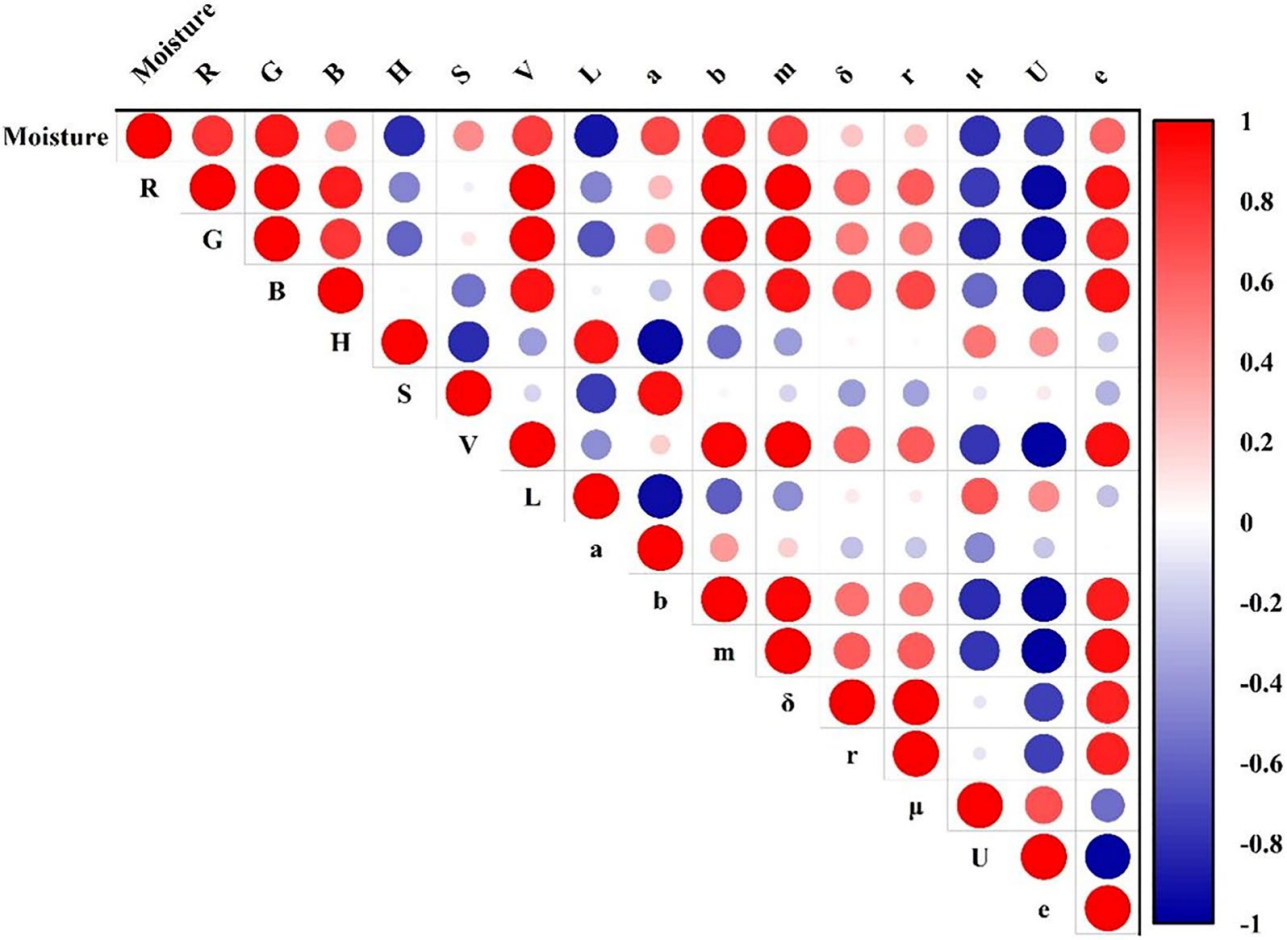
The selected effective features for image data.

Regarding the image information of withering samples, Pearson correlation analysis was used to obtain the variables, which were highly correlated with moisture. Figure 5 displays the Pearson correlation analysis between 15 color, texture and moisture content features. All 15 variables were significantly correlated with the moisture content of withering leaves because their p values were lower than 0.01. Nevertheless, not all color and texture features displayed a high correlation with the change in moisture content. The correlation coefficient for R, G, B, H, S, V, L, a, b, m, δ, r, μ, U and e was 0.79, 0.89, 0.46, − 0.80, 0.45, 0.76, − 0.89, 0.72, 0.87, 0.76, 0.24, 0.25, − 0.78, − 0.77, and 0.6, respectively. Generally, variables with high correlation coefficients should be retained, otherwise they should be removed. In this research, the variable was selected when the value of correlation coefficient was higher than 0.6. Accordingly, 7 color and 4 texture features, such as R, G, H, V, L, a, b, and m, μ, U and e, respectively, were selected for the middle-level fusion strategy.

### Moisture prediction models using a single technology

The SVR models were applied to predict the moisture content of withering leaves using a single technology such as NIRS or E-eye. The results were displayed in Table 1; for the single E-eye sensor, the R_c_, RMSEC, R_p_, RMSEP, and RPD were 0.9935, 0.0116, 0.9823, 0.0216, and 4.6491. Both the calibration and prediction set showed a satisfactory performance. For the single NIRS sensor (removed noise bands), the R_c_, RMSEC, R_p_, RMSEP, and RPD values were 0.9969, 0.0081, 0.9788, 0.0204, and 4.7213, respectively. The established model using NIRS data was compared with the model using E-eye data. For the prediction set, although the E-eye model had a higher R_p_ value, this model also had a higher RMSEP value and lower RPD value. Importantly, the model using NIRS data had a higher RPD value, indicating that it achieved more satisfactory accuracy and more applicable. Hence, it could more precisely predict the moisture content of withering leaves. Remarkably, the NIRS technology could predict the moisture content based on the internal information of withering leaves, such as the characteristics of special groups. Nevertheless, the E-eye technology could obtain the external information including color and texture features. The performance of the model using spectral data was slightly better than that using color and texture data, because the color and texture features could not comprehensively express the changes in the moisture level of withering leaves. Therefore, the internal and external information of withering leaves should be combined for further analysis.

### Moisture prediction models with multilevel data fusion

For different sensors, the implementation of a data fusion strategy is particularly important. In this study, the spectral, color and texture information were collected for low- and middle-level fusion strategy. Although the low-level fusion strategy obtained the most comprehensive data information, it could retain a lot of redundant information. Therefore, in the middle-level fusion strategy, PCA was applied to compress the variable information. In addition, RF and Pearson correlation analysis were applied to select effective bands and appearance features for spectral and image data, respectively. Finally, the SVR algorithm was used to establish the moisture detection model.

#### Low-level fusion strategy

In order to improve the comprehensiveness of the obtained information and the accuracy of the established moisture detection model, the low-level fusion strategy was performed. The preprocessed spectra and appearance features (color and texture features) were simply concatenated into a new matrix, which was normalized and applied to establish the moisture detection model. The results were shown in Table 2. The R_c_, RMSEC, R_p_, RMSEP, and RPD values were 0.9931, 0.0117, 0.9872, 0.0182, and 5.5937, respectively. Compared with the moisture detection model using single technology, the model using low-level strategy exhibited a better prediction performance. For the NIRS and E-eye technology, the RPD values were increased by 0.8707 and 0.9446, respectively. Although the low-level fusion strategy showed a satisfactory result, the collected information suffered from high data dimensionality, and the running speed would be affected. Hence, the data fusion strategy of feature level should be applied to establish the moisture prediction model.

**Table 2:** SVR models for moisture content detection in withering leaves using multi-level data fusion.

Level	Methods	No. of variables	Parameter		Calibration sets		Prediction sets		
			c	g	R_c_	RMSEC	R_p_	RMSEP	RPD
Low-level	PCA	760	0.4353	256	0.9931	0.0117	0.9872	0.0182	5.5937
		13	0.3299	0.0206	0.9939	0.0112	0.9849	0.0201	5.0295
Middle-level	RF	112 (cutoff = 0.6)	1.3195	256	0.9969	0.0081	0.9780	0.0226	4.3343
		44 (cutoff = 0.7)	6.9644	256	0.9965	0.0084	0.9851	0.0199	5.0222
		19 (cutoff = 0.8)	0.25	256	0.9912	0.0133	0.9883	0.0180	5.5596

#### Middle-level-PCA fusion strategy

**Figure 6:**
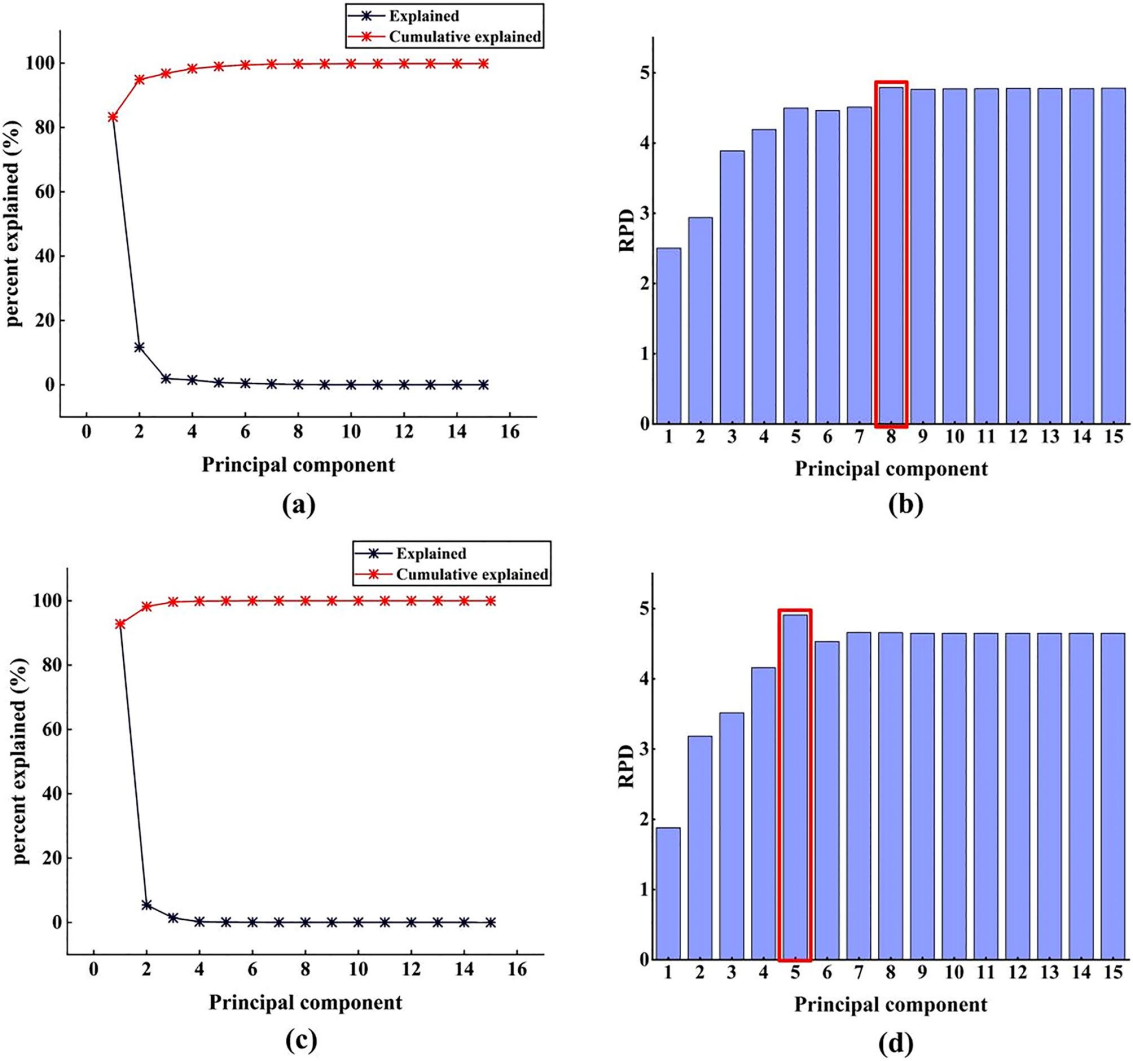
Percentage and cumulative spectral data (**a**) and image data (**c**) explained by PCA; SVR prediction models for different numbers of PCs to determine the best RPD value for spectra data (**b**) and image data (**d**).

For the middle-level-PCA strategy, the spectral data and appearance features were subject to PCA. The explained and the cumulative explained percent for the spectral data and image data using the first 15 PCs were displayed in Fig. 6a,c, respectively. For the spectral data, the first 15 PCs could represent 99.86% of the information in raw tea samples. For the image data, the first 15 PCs could represent 100% of the information in raw tea samples. Subsequently, the RPD values of the established models for the spectral data and image data using the first 15 PCs were shown in Fig. 6b,d, respectively. As can be seen from the figures, the established SVR models for spectral data and image data obtained the optimal RPD values when the PCs were 8 and 5, respectively. After the selection of optimal RPD values, the first 8 PCs of spectral data and first 5 PCs of image data were directly connected. Then, the calibration set and prediction set were divided using the Kennard-Stone method with a ratio of 3:1 to establish the moisture detection model. The results were displayed in Table 2. Compared with either single technology, the middle-level-PCA strategy improved the prediction performance of the model because the R_p_ value and RPD value were improved. At the same time, although the variable compression ratio could reach 98.29%, indicating that the running speed of the prediction model was greatly improved, the accuracy of the moisture prediction model slightly decreased compared to the low-level strategy.

#### Middle-level-RF fusion strategy

For the middle-level-RF strategy, the RF algorithm and Pearson correlation analysis were used to select effective bands and appearance features, respectively. The selected appearance features, including 7 color and 4 texture features, included R, G, H, V, L, a, b, and m, μ, U, e, respectively. In the RF strategy, three cutoff lines were determined and the effective bands were selected. Subsequently, the SVR model was established using the fusion data of middle-level-RF strategy to predict the moisture content in withering samples. The selected effective features using the middle-level-RF strategy and the model performance were displayed in Fig. 4 and Table 2, respectively. When the cutoff line was set as 0.6, a total of 112 effective features (101 bands and 11 appearance features) were selected, and the variable compression rate was 85.26%. The R_p_, RMSEP and RPD values were 0.9780, 0.0226 and 4.3343, respectively. When the cutoff line was set as 0.7, a total of 44 effective features (33 bands and 11 appearance features) were selected, and the variable compression rate was 94.21%. The R_p_, RMSEP and RPD values were 0.9851, 0.0199 and 5.0222, respectively. When the cutoff line was set as 0.8, a total of 19 effective features (8 bands and 11 appearance features) were selected, and the variable compression rate was 97.50%. The R_p_, RMSEP and RPD values were 0.9883, 0.0180 and 5.5596, respectively. With the increase in the values of the cutoff line, the compression rate of the value and the accuracy of the model also gradually increased, indicating that the change of the cutoff line made a great contribution to the compression rate of the value, and there was a considerable amount of redundant information in the raw spectral data that could affect the accuracy of the moisture detection model. In general, the most moisture detection model using the middle-level fusion strategy displayed satisfactory results.

### Discussion

According to the above analysis, the established models based on single technology and different data fusion strategies were compared. The most moisture prediction models using a data fusion strategy, except for the middle-level-RF (cutoff line = 0.6) strategy, showed a better performance than the model using a single technology. Obviously, the middle-level-RF (cutoff line = 0.6) strategy displayed the worst performance, showing that only an appropriate data fusion strategy could improve the performance of the moisture detection model. The low-level fusion strategy and the middle-level-RF (cutoff line = 0.8) strategy displayed similar prediction results. However, in contrast to the low-level fusion strategy, the middle-level-RF (cutoff line = 0.8) strategy used fewer variables to build the moisture detection model and still achieved a satisfactory result. In actual production, data collection time and model response speed are important reference factors. As indicated by the results, the middle-level-RF (cutoff line = 0.8) required shorter data collection time and achieved faster model response speed. Hence, the model using the middle-level-RF (cutoff line = 0.8) strategy was considered to be the best moisture detection model. During the withering process, the stems and leaves undergo gradual wilting and the color of the leaves change from light green to dark green. For the color and texture features, 11 variables, including R, G, H, V, L, a, b, m, μ, U, and e, with high correlation coefficients were selected, whereas 4 variables, such as B, S, δ, and r, were removed because they were insensitive to the changes in leaf external features. For the spectral data, the middle-level-RF (cutoff line = 0.8) strategy selected 8 effective bands, including 936 nm, 988 nm, 989 nm, 1023 nm, 1211 nm, 1241 nm, 1267 nm, and 1298 nm, and these bands might be related to the second OH- and CH-stretching overtone spectra^32,33^. In the withering process of black tea, the moisture loss is very obvious and is also accompanied by enzymatic reactions and the reduction of compounds with hydrogen. Hence, in addition to moisture, some key components, such as amino acids, soluble sugars and catechins, change significantly^34^. These selected bands using the middle-level-RF strategy could be directly or indirectly used as a basis for moisture content evaluation.

Although both the NIR and E-eye model showed satisfactory performance, there are some shortcomings in NIR and E-eye technology. For instance, the NIR technology could only obtain the information in a very small range of samples because of its optical fiber. However, in actual production, the spreading area of withering samples is large. Compared with the NIR technology, the E-eye technology could obtain a wide range of sample information. However, the E-eye technology still has shortcomings. This technology could only obtain the information on the surface of withering samples and has no penetrability to the withering leaves. In contrast to the E-eye technology, the NIR technology could show certain penetrability. Hence, the obtained information by a single technology is one side and they could be influenced by uneven withering. Therefore, it is necessary to fuse spectral and image information.

Liang et al.^9^ and Zhang et al.^35^ established two kinds of models for the moisture prediction of tea leaves using E-eye and NIRS, respectively. Although these models were reliable, our proposed moisture detection model using middle-level-RF (cutoff line = 0.8) fusion strategy presented higher accuracy. Liu et al.^15^ compared data fusion strategies to predict the moisture content during green tea processing; the middle-level-SVR strategy displayed the best performance, and the R_p_, RMSEP and RPD values were 0.9777, 0.0490 and 4.5002, respectively. Apparently, our proposed middle-level-RF (cutoff line = 0.8) fusion strategy showed a more satisfactory result. Hence, the model using our proposed data fusion strategy could more accurately evaluate the moisture of withering leaves.

According to the above discussion, the innovations of this study are as follows: (1) the fusion information of spectra and image is used to establish the moisture prediction model for black tea withering leaves. (2) The different cutoff lines based on RF method were used to select effective spectral information and these selected information were fused with effective image information to establish the moisture prediction model. (3) The selection of color and texture features using Pearson correlation analysis.

## Conclusions

This study proved the feasibility of moisture level prediction in withering leaves using NIRS combined with E-eye technology. Different data fusion strategies were applied and compared in tandem with the SVR quantitative prediction models. It was demonstrated that an appropriate data fusion strategy could improve the performance of the moisture detection model for withering leaves. In addition to the middle-level-RF (cutoff line = 0.6) strategy, the performance of models using other fusion strategies were better than the model using a single technology. Moreover, the middle-level-RF (cutoff line = 0.8) strategy used fewer variables to build the moisture prediction model and still achieved a satisfactory result, with 0.9883, 5.5596 and 97.50% for the R_p_, RPD and the variable compression rate, respectively. Therefore, our proposed data fusion strategy can be used as a theoretical reference for the precise control of black tea withering.

Although the strategy described herein could effectively improve the performance of the model, there are some shortcomings to be improved. Accordingly, some ideas for future studies are given: (1) Withering experiments involving different varieties and seasons should be carried out to improve the robustness of the established moisture detection model. (2) New equipment that can simultaneously obtain the information of image and spectra for withering leaves should be developed.

## Data Availability

The datasets generated and/or analysed during the current study are not publicly available due the data would be used to develop equipment with integrated functions but are available from the corresponding author on reasonable request.
